# "More money for health - more health for the money": a human resources for health perspective

**DOI:** 10.1186/1478-4491-9-18

**Published:** 2011-07-15

**Authors:** James Campbell, Iain Jones, Desmond Whyms

**Affiliations:** 1 Instituto de Cooperación Social, Integrare (ICSI), Barcelona, Spain; 2 Economic Adviser, Department for International Development (DFID), London, UK; 3 Senior Health Adviser, DFID, London, UK

## Abstract

**Background:**

At the MDG Summit in September 2010, the UN Secretary-General launched the Global Strategy for Women's and Children's Health. Central within the Global Strategy are the ambitions of "more money for health" and "more health for the money". These aim to leverage more resources for health financing whilst simultaneously generating more results from existing resources - core tenets of public expenditure management and governance. This paper considers these ambitions from a human resources for health (HRH) perspective.

**Methods:**

Using data from the UK Department for International Development (DFID) we set out to quantify and qualify the British government's contributions on HRH in developing countries and to establish a baseline.. To determine whether activities and financing could be included in the categorisation of 'HRH strengthening' we adopted the Agenda for Global Action on HRH and a WHO approach to the 'working lifespan' of health workers as our guiding frameworks. To establish a baseline we reviewed available data on Official Development Assistance (ODA) and country reports, undertook a new survey of HRH programming and sought information from multilateral partners.

**Results:**

In financial year 2008/9 DFID spent £901 million on direct 'aid to health'. Due to the nature of the Creditor Reporting System (CRS) of the Organisation for Economic Co-operation and Development (OECD) it is not feasible to directly report on HRH spending. We therefore employed a process of imputed percentages supported by detailed assessment in twelve countries. This followed the model adopted by the G8 to estimate ODA on maternal, newborn and child health. Using the G8's model, and cognisant of its limitations, we concluded that UK 'aid to health' on HRH strengthening is approximately 25%.

**Conclusions:**

In quantifying DFID's disbursements on HRH we encountered the constraints of the current CRS framework. This limits standardised measurement of ODA on HRH. This is a governance issue that will benefit from further analysis within more comprehensive programmes of workforce science, surveillance and strategic intelligence. The Commission on Information and Accountability for Women's and Children's Health may present an opportunity to partially address the limitations in reporting on ODA for HRH and present solutions to establish a global baseline.

## Background

At the MDG Summit in September 2010, the United Nations Secretary General (UNSG) launched the Global Strategy for Women's and Children's Health [1]. The strategy sets out the key areas where action is urgently required to enhance financing, strengthen policy and improve service delivery. It represents, in the UNSG's own words, an opportunity "to improve the health of hundreds of millions of women and children around the world, and in so doing, to improve the lives of all people" [2]. Central within the Global Strategy are the ambitions of "more money for health" and "more health for the money".

**Figure 1:**
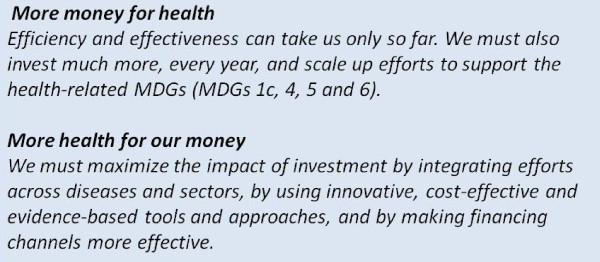
**"More money for health - more health for the money"**. Source: Global Strategy for Women's and Children's Health [3].

The objectives aim to leverage "more" resources and "more" results. They refer to the additional financing required to achieve the Millennium Development Goals for health ("spending on health in low-income countries needs to be raised from an estimated US$ 31 billion [in 2009] to US$67-76 billion per year by 2015" (more money for health)) and the necessity to improve the use of existing financial resources to strengthen health systems and scale-up efficient, effective and equitable services that result in improved health outcomes (more health for the money). Both are core tenets of public expenditure management and governance; equally applicable to domestic and international expenditures (see Figure 1).

This paper responds to the two ambitions in the UNSG's Global Strategy from a human resources for health (HRH) perspective. It draws upon formative monitoring and evaluation activities within the United Kingdom of Great Britain and Northern Ireland (United Kingdom) Department for International Development (DFID) to quantify and qualify the British Government's support to HRH. To paraphrase the Global Strategy the paper reviews issues related to "more HRH for the money" and "more money for HRH". A key purpose of the research was to address the feasibility of establishing a baseline from which to measure 'more'.

The paper is presented in three parts. In the first we describe the methodology employed in establishing a baseline. The second part presents a short overview of the results before focusing on the quantitative component related to Official Development Assistance (ODA) for HRH. This leads to a discussion, drawing on the peer-reviewed literature, of the OECD's Creditor Reporting System (CRS) in relation to HRH strengthening in the final part.

## Methods

**Figure 2:**
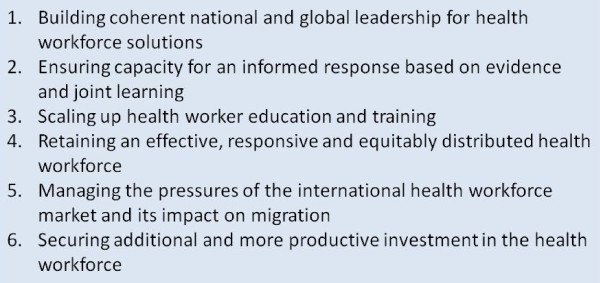
**Six action areas from the Agenda for Global Action on HRH**. Source: Global Strategy for Women's and Children's Health [3].

**Figure 3:**
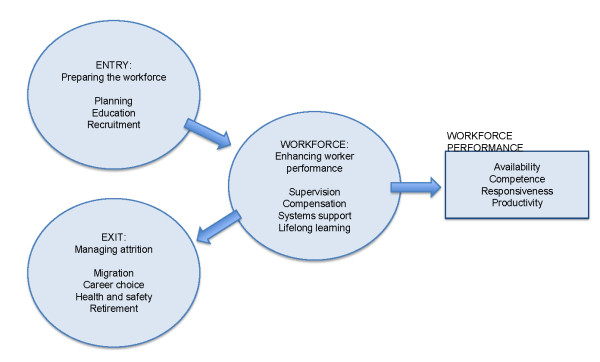
**WHO: Working lifespan strategies**. Source: World Health Report 2006 [4]

In order to determine whether activities and financing could be included in the categorisation of 'HRH strengthening' we adopted two guiding frameworks: the *Agenda for Global Action on HRH *[3] (see Figure 2) and WHO's approach to the working lifespan of health workers [4] (see Figure 3). The *Agenda for Global Action on HRH *and the accompanying *Kampala Declaration *[5] were prepared by the Global Health Workforce Alliance (GHWA) in 2008. These have since been recognised by the G8 as tools to guide collective action [6,7]. Comparing British-funded activities against the Agenda for Global Action served a dual purpose: to be one of the first bilateral agencies to classify British activities against each of the six action areas in the Agenda (thus evaluating whether UK programming is consistent with this widely-adopted consensus for action on HRH) and for subsequent internal and external reporting (i.e. for reporting UK activities on HRH to the G8 as required by their annual Accountability Framework). The World Health Organization (WHO) 'working lifespan strategies' is promoted as a roadmap for training, sustaining and retaining the workforce [4] and provided a visual tool to assess and categorise UK-supported activities (see Figure 2 and 3).

Three components were included in the research: a desk-based analysis of ODA, an in-depth review in four countries and a survey of HRH programming across twelve countries.

We conducted a desk-based analysis of the British ODA in the 2008/9 financial year to quantify the volume and percentage of DFID spending on 'aid to health' that was committed to HRH strengthening across all countries. We analysed data from the 2008/9 financial year (FY) (the most recent and complete for both multilateral and bilateral sector spending) to base our assessment on ODA disbursements rather than projections, extracting data from DFID's management information system. This relational database disaggregates health expenditure by sector and sub-sector codes as per the Creditor Reporting System (CRS) of the Organisation for Economic Co-operation and Development (OECD). Due to limitations in the coding structure of the CRS we were aware that total volumes and percentages could not be calculated purely by summing the specific sub-sector codes for HRH activity. Instead we elected to calculate rational estimates on the HRH expenditures within other sub-sector codes. These rational estimates followed a process of imputed percentages, mirroring the exercise developed by G8 partners to assess and benchmark ODA for maternal, newborn and under-five child health (MNCH) [8]. The MNCH exercise was undertaken in preparation for the G8 Statement in June 2010 announcing the Muskoka Initiative on MNCH [9]. It provided an estimate of G8 spending on MNCH (with supporting rationale), overcoming the limitations of the Creditor Reporting System, and a baseline for future accountability mechanisms (see Table 1).

**Table 1:** G8 Health Working Group - imputed percentages for bilateral expenditure on MNCH

DAC CRS Code		Imputed Percentages
12110	Health policy and administrative management	40%

12181	Medical education/training	40%

12191	Medical services	40%

12220	Basic health care	40%

12230	Basic health infrastructure	40%

12240	Basic nutrition	100%

12250	Infectious disease control	40%

12261	Health education	40%

12262	Malaria control	88.5%

12263	Tuberculosis control	18.5%

12281	Health personnel development	40%

13010	Population policy and administrative management	40%

13020	Reproductive health care	100%

13030	Family planning	100%

13040	STD control including HIV/AIDS	46.1%

13081	Personnel development for population and reproductive health	100%

14030	Basic drinking water supply and basic sanitation	15%

14031	Basic drinking water supply	15%

14032	Basic sanitation	15%

51010	General budget support	4%

Source: Adapted from G8 Information Centre, University of Toronto. http://www.g7.utoronto.ca/summit/2010muskoka/methodology.html

The process of imputed percentages for estimating a specific type of spending from a sub-sector code is one with methodological limitations. Being cognisant of the huge challenges in measuring ODA we differed from the G8's MNCH exercise in Table 1 by electing to use a range in our rational estimates of 10% (low and high being +/-5%) to estimate an approximate value. Each estimate of HRH spending in sub-sector codes was based on the data and trends emerging from the detailed analysis of the individual country portfolios (components 2 and 3 of the research discussed below). We compared technical activities and financial allocations within and across country programmes to estimate the volume of funds for HRH strengthening. DFID colleagues were subsequently invited to challenge the rationale and logic in our estimates. In some instances our estimates were revised downwards to err on the side of caution. We also tested the estimates and resulting average against total 'aid to health' spending in previous financial years (2005-6, 2006-7 and 2007-8) to assess if this would significantly change over more than one financial year, and found this not to be the case.

In support of the ODA exercise the research included two further components to qualify British-supported activities and to develop and test our rationale for the imputed percentages in the sub-sector codes. Four countries had earlier participated in an in-depth analysis of HRH programming as part of the United Kingdom's joint work on 'Taking Forward Action on HRH' with the USA's President's Emergency Programme for AIDS Relief (PEPFAR). The four countries were selected on the basis of being signatories to the International Health Partnership and related initiatives (IHP+) and 'focus' countries for PEPFAR at that time. These studies were conducted jointly with the Ministries of Health in the respective countries and the USA's Office of the Global AIDS Coordinator (OGAC). Reviews were undertaken in Ethiopia, Kenya, Mozambique and Zambia in the period 2008-9. Key informant interviews and focus groups were combined with desk reviews of technical and financial documentation to summarise existing HRH strengthening activities and discuss future opportunities for enhanced programming and alignment [10-14].

The third component was a multi-country survey in the latter half of 2009. We invited DFID's residential health advisers in 22 priority countries to relate DFID's investments in HRH strengthening (including general budget support, sector support and direct programming) to the six recommended action areas in the Agenda for Global Action on HRH. Country health advisers completed a standardized questionnaire to identify technical activities and financial spending. This facilitated a detailed assessment of ODA for HRH and enabled a comparison against the coding of expenditure in DFID's internal system. Country returns were reviewed and, where required, clarification questions were conducted by telephone and/or email. Response rates (n = 12) from DFID's country advisors determined the inclusion of countries in the survey.

The three components provided a rich data set for internal analysis. Twelve countries--Bangladesh, Cambodia, the Democratic Republic of Congo, Ethiopia, Ghana, India, Kenya, Mozambique, Nigeria, Sierra Leone, South Africa, Zambia and Zimbabwe (South Africa being the only country among these not categorised as an HRH 'crisis' country)--from DFID's portfolio of development support participated in the country visits and/or the multi-country survey (nine from sub-Saharan Africa, three from South-East Asia). The data on financial programming and disbursements enabled rational estimates to be made for the imputed percentages on ODA. Initial findings were synthesised and discussed prior to scrutiny and internal review from DFID colleagues to inform future programming.

## Results

Of the twelve countries, eleven are listed as having a critical shortage of health workers in the 2006 World Health Report. Density of health professionals (doctors, nurses and midwives per 1000 population) in the eleven countries is in the range of 0.25 to 2.13/1000, as against the threshold of 2.28/1000, below which WHO has suggested that high coverage of essential interventions, including skilled attendance at birth, is very unlikely. The sum of the estimated health workforce shortages in these eleven 'crisis' countries is 2.1 million, or half of the global shortage of 4.2 million [4].

In FY 2008/9, the latest available data for both multilateral and bilateral sector disbursements, DFID spent £901 million on direct 'aid to health'. This was approximately a 75:25 split through bilateral and multilateral channels [15]. Of note is that 56% of DFID's bilateral health spending in 2008/09 was disbursed to the eleven countries highlighted above. This confirms that just over one half of the UK's bilateral support is targeted to those HRH 'crisis countries' that exhibit one-half of the global workforce shortage and provides a weighted sample for the rationale underpinning the imputed percentages.

### ODA for HRH strengthening

Reflecting item 6 in the Agenda for Global Action, to secure 'additional and more productive investment in the health workforce', we set out to quantify the baseline of current HRH spending across both bilateral and multilateral channels. The CRS collates aid flows at activity level. Two sector codes, 'health' and 'population policies/programmes and reproductive health', are sub-divided by seventeen sub-sector codes. Collectively these are considered 'aid to health' [16,17]. DFID's internal management information system, known as "ARIES", follows the CRS sector and sub-sector coding to facilitate statistical and annual reporting on ODA. Within ARIES these are referred to as 'input sector codes'.

In tracking the bilateral spending, an immediate difficulty arises in reporting ODA committed to HRH strengthening. Of the 17 codes for 'aid to health' (note there are other OECD codes relating to public sector policy and management, which are not traditionally 'aid to health' but which may also capture spending related to human resource management) only 3 - the "81's" - provide specific wording related to education/training and personnel development: 12181: Medical education/training; 12281: Health personnel development; 13081: Personnel development for population and reproductive health. Of these, one is only for activities supporting tertiary services (12181: medical education/training). These 3 codes are not representative of the breadth and depth of DFID's current HRH programming or the recommended activities in the Agenda for Global Action. Reporting HRH spending based only on the figures captured in these three sub-sector codes would generate figures of little value as well as obscuring the more complex reality of HRH strengthening recognised by Piva and Dodd (2009) [18]

This dilemma is recognised by DFID's internal system. It allows up to eight input sector codes to be assigned to capture the multiple elements of health programming. Where more than one code is indicated, then the proportion of the lifetime budget expected to be spent in each sub-sector must be indicated as a percentage, and the total must sum to 100%. This system provisionally enables disaggregated data to more closely reflect the actual investments.

However, even disaggregated data by input sector code may still require an assessment of the percentage of funds dedicated to HRH. For instance, the UK is providing £135 million to Ethiopia in pooled-funds for 'Protecting Basic Services'. A WHO study notes that Ethiopia's Health Extension Program (HEP) particularly benefits from this programme, and around 6-7% (USD 72-84 million) of the first phase of the pooled-funding was used for direct salary support for health workers [19]. In this particular example, DFID's investment is recorded against Poverty Reduction Budget Support (attributed to health); basic health care; infectious disease control; and reproductive health care. Even with disaggregation, the HRH spend still remains obscured.

Offsetting these coding and categorisation issues therefore requires a detailed understanding of context. This was provided by the qualitative components of the research and enabled the construction of estimated percentages supported by rational assumptions. Continuing with the Ethiopia example, DFID funding supports the government's recurrent costs, the rapid expansion of the health workforce and salary support. This includes the training and deployment of 30 000 health extension workers. In this instance we estimated that a range of 25%-30% of the budget support may be indirectly strengthening HRH. Whilst this 'rational' approach may provide greater insights into the realistic volume of HRH investment, we have to recognise its methodological weaknesses and provide caveats alongside any final estimates.

Estimating the percentages and volumes of multilateral expenditure on HRH comes up against similar problems to the bilateral expenditures (the channels include core contributions to multilateral agencies and specific commitments to the Global Fund for AIDS, TB and Malaria (GFATM), the Global Alliance for Vaccines and Immunisation (GAVI) and the International Financing Facility for Immunisation (IFFIm)). While we could calculate a three-year average of the multilaterals' or global partnerships' disbursement data as reported to the CRS it may only reflect the 3 specific codes (assuming data is captured at this disaggregate level) and not the wider HRH activities. We therefore sought to review existing documentation to provide the rationale for our estimates, accepting that the principle of the 'primary' code in OECD aggregate reporting masks the commitment to HRH. Our focus was on the European Commission, the World Bank and the Global Fund for AIDS, TB and Malaria (GFATM) as the three largest recipients of DFID's multilateral health investments.

We first queried the CRS database records (using the Query Wizard for Information on Development Statistics; accessed 10 March 2010) for the World Bank and the GFATM (the EC was not included as there is limited disaggregated data for its 'aid to health'). No disbursements on the three specific sub-sector codes - the "81's" - are indicated by the Bank in their 2008 data and equally no data is reported by the GFATM in the period 2003-2008. For the Global Fund this is at odds with their 2009 narrative that it has supported 8.6 million "person episodes" of training since 2004 [20]. Equally, HRH strengthening is evident in the Global Fund's cross-cutting health systems strengthening activities (including direct salary support to health workers), many of the approved country applications and the Fund's own statistics. However, GFATM reports offer differing analysis and information on how much it commits to HRH investment and activities. The 2009 report suggests that 35% of all funding has been for systems strengthening, including increasing the number, skills and competencies of health workers. Meanwhile a survey across 65% of its active portfolio in 2007 indicated that 25% of all funds are allocated to human resources and training, and 42% of all activities in Board-approved Round 8 proposals related to human resources and training [21]. The various interpretations of the core data, without specific attention to actual HRH investment as a percentage of total spending and without year-on-year comparison combine to confuse.

Drawing little information from the CRS we therefore requested feedback directly from the European Commission (EC), the World Bank and the GFATM. The EC was unable to provide an estimate but did qualify current expenditure within their Programme for Action on HRH [22] and related activities under the Investing in People budget line. Indicative figures on HRH spending were provided as a percentage of aid to health expenditure in the last five years by the World Bank and the GFATM. These were 18% and 21% respectively. The Bank's indicative estimate came from a sample of approximately 30 programmes. The Global Fund's 21% figure relates to an HRH investment of circa $1.5 billion in Rounds 5 to 9. Whilst both these figures have to be treated with the same appropriate caution as DFID's internal estimates of its bilateral investment, they nonetheless provided some external assessment to work with.

Table 2 presents the final calculations on estimated spending on HRH strengthening, including bilateral and multilateral channels. The imputed percentages from our representative sample when applied across the total 'aid to health' indicate the volume of ODA to HRH is in the range of £200-£285 million (equivalent to a low of 22% and a high of 32%). For internal purposes we therefore concluded with a working figure in the lower half of the range of 'approximately 25%' (See Table 2).

**Table 2:** DFID: ODA on HRH strengthening - imputed percentages

Code	Activities	Description	Allocation (LOW)	Allocation (HIGH)	£ (,000) 2008/09	Estimate (LOW) £ (,000)	Estimate (HIGH) £ (,000)
***Direct Activities ***							

13010	Population policy and administrative management: Health	Population/development policies; census work, vital registration; migration data; demographic research/analysis; reproductive health research; unspecified population activities.	15%	25%	2,619	393	655
13021	Reproductive health care	Promotion of reproductive health; prevention and treatment of infertility;	25%	35%	36,466	9,116	12,763
13022	Maternal and neonatal health	Prenatal and postnatal care including delivery; prevention and management of consequences of abortion; safe motherhood activities.	25%	35%	61,645	15,411	21,576
13030	Family planning, health	Family planning services including counselling; information, education and communication (IEC) activities; delivery of contraceptives; capacity building and training.	25%	35%	8,075	2,019	2,826
13081	Personnel development for population and reproductive health	Education and training of health staff for population and reproductive health care services.	100%	100%	1,490	1,490	1,490
12010	Health Poverty Reduction Budget Support	Attributed PRBS to the health sector	20%	30%	105,679	21,136	31,704

***Indirect Activities***							

12110	Health policy and Administrative management	Health sector policy, planning and programmes; aid to health ministries, public health administration; institution capacity building and advice; medical insurance programmes; unspecified health activities	25%	35%	48,784	12,196	17,074
12220	Basic health care	Basic and primary health care programmes; paramedical and nursing care programmes; supply of drugs, medicines and vaccines related to basic health care.	20%	30%	99,652	19,930	29,896
12240	Basic nutrition, Health	Direct feeding programmes (maternal feeding, breastfeeding and weaning foods, child feeding, school feeding); determination of micro-nutrient deficiencies; provision of vitamin A, iodine, iron etc.; monitoring of nutritional status; nutrition and food hygiene education; household food security.	15%	25%	12,927	1,939	3,232
12261	Health education	Information, education and training of the population for improving health knowledge and practices; public health and awareness campaigns.	25%	35%	19,842	4,961	6,945
12262	Malaria control	Prevention and control of malaria	25%	35%	35,060	8,765	12,271
12281	Health personnel development	Training of health staff for basic health care services.	100%	100%	10,918	10,918	10,918
13041	HIV/AIDS including STD prevention	Activities related to prevention of sexually transmitted diseases and HIV/AIDS e.g. information, education and communication; testing; prevention;	35%	45%	147,863	51,752	66,538
13042	HIV/AIDS including STD Treatments and Care	Activities related to treatment and care of sexually transmitted diseases and HIV/AIDS	35%	45%	10,113	3,540	4,551

***Research***							

80012	Health Research		15%	25%	48,900	7,335	12,225

***Multilateral and vertical funds***							

	DFID health aid through:	EC	15%	25%	1,153,892	5,279	8,798
		World Bank	13%	23%	573,652	8,007	14,166
		AfDB	15%	25%	139,000	263	438
		AsDB	15%	25%	28,534	177	296
		UNAIDS	15%	25%	10,000	1,500	2,500
		UNICEF	15%	25%	16,000	325	542
		World Health Org	15%	25%	12,500	1,875	3,125
		UN Population Fund	15%	25%	20,000	3,000	5,000
		UNDP	15%	25%	55,000	257	428
		IFFIm (4)	0%	10%	16,849	0	1,685
		GAVI	15%	25%	0	0	0
		GFATM (3)	16%	26%	50,000	8,000	13,000

			**TOTAL ODA to HRH**			**199,584**	**284,642**

			BILATERAL ODA		684,931		
			MULTILATERALODA		216,403		
			TOTAL ODA		901,335		

		**PERCENTAGE of ODA to HRH**				**22%**	**32%**

Source: Global Health Workforce Alliance. Kampala Declaration and Agenda for Global Action. Authors calculations. Adapted from SID.

http://www.dfid.gov.uk/Documents/publications1/sid2010/table20.xls?epslanguage=en

http://www.dfid.gov.uk/Documents/publications1/sid2010/a3.xls?epslanguage=en

## Discussion

This research was developed to provide strategic intelligence for internal discussion within DFID. An informed baseline on HRH activities would support the exploration of future programming and financing scenarios as the UK developed its 2011-2015 programme of aid to health. Additionally the results would be available for discussion with partners and civil society and in responses to British parliamentary questions [23,24].

It was conducted against a backdrop of international commitments to meet development spending targets, increasing attention to results, value-for-money, the 'Decade for Action on HRH' called for in the 2006 World Health Report and revised projections on the financing needs for the health MDGs in the lead up to the 2010 MDG Summit. A key consideration was the UK Government's commitment to meeting the target of 0.7% of Gross National Income on development spending by 2013. Deputy Prime Minister Nick Clegg's speech at the United Nations General Assembly in September 2010 outlined this commitment, emphasising the accountability for targeted investments and results:

"So my message to you today, from the UK government, is this - we will keep our promises; and we expect the rest of the international community to do the same. For our part, the new coalition government has committed to reaching 0.7% of GNI in aid from 2013 - a pledge we will enshrine in law. That aid will be targeted in the ways we know will make the biggest difference" [25].

The UK messaging on "more resources" and "more results" was further articulated by the Secretary of State Andrew Mitchell in October 2010: "...we have a particular duty to show that we are achieving value for money. Results, transparency and accountability will be our watchwords and will define everything we do" [26].

This emphasis on results and enhanced accountability is not restricted to the UK Government. The UNSG's Global Strategy and the outcome document from the MDG Summit both recognise this. A recently established Commission on Information and Accountability for Women's and Children's Health [27] highlights this even further. Of note is that the two working groups convened by the Commission are respectively focused on 'Accountability for Resources' and 'Accountability for Results'. The same principles of accountability and transparency are inherent in the aid effectiveness agenda [28] and explicit in the Centre for Global Development's recent Report on the Quality of Official Development Assistance Assessment (QuODA) [29].

Applying the same considerations to HRH strengthening was therefore a logical extension of this emphasis. The results demonstrate that DFID is supporting an active HRH portfolio working with national partners across the range of priority actions recommended in the Agenda for Global Action. This includes developing capacity for human resource management, expansion of pre-service education and initiatives to support rural deployment and retention: essential elements to get the right health worker in the right place at the right time. In tackling the quantification of ODA for HRH the internal exercise raised a number of issues that are relevant to a wider external audience. These are explored further below.

The difficulties in conducting detailed analysis of 'aid to health' or sub-sectors of this is an acknowledged issue [18,30-32]. It is not unique to HRH. However, in narrowing the focus to ODA for HRH, we have identified a number of issues. These highlight the methodological challenges to assess and routinely measure donors' investments in HRH strengthening.

Firstly, there is a major disconnect between disbursements on HRH and the creditor reporting system. The current reporting framework, described by WHO as ill-adapted to isolating HRH expenditures [33], results in the statistic of less than 4% of "aid to health" being dedicated to training and personnel development. Whilst the OECD acknowledges that training is itself only a small part of workforce development and dramatically understates the workforce strengthening activities of donors it concedes that the real share of ODA to HRH cannot be identified [17]. Conversely, Chen et al. (2004) as part of the landmark Joint Learning Initiative report on HRH estimated that somewhere between "30-50% of ODA is devoted to human resources--salaries, allowances, training, education, technical assistance, and capacity building" [31]. This range in estimates, from the OECD's 4% to the JLI's upper figure of 50%, clearly demonstrates a major flaw in the current system for standardised reporting.

Additionally, the CRS coding encourages most HRH-related investment to be 'hidden' and 'obscured'. The CRS coding focuses on education/training and personnel development. These are essential elements of workforce development but do not reflect the WHO understanding of HRH across the working lifespan strategies. By default, all other HRH related investments are 'hidden' in other sector codes. Due to the system of ODA reporting on aggregate data, these are then obscured further. It is only the 'primary sector' - i.e. the code with the greatest percentage of the financing - which is referenced in reports. The example of the Global Fund, where $1.5 billion of HRH spending is not clearly evident, is perhaps the most striking example. Whilst this avoids double-counting, the downside is that this classification and aggregate reporting results in an 'all-or-nothing' situation [34]. Dodd et al. (2009) have found similar difficulties in ODA reports and how to disaggregate HRH expenditure in Lao PDR [35].

Further, it is unlikely that data is being captured and reported with the same consistency across programming and agencies. With a limited choice of codes reflecting HRH investments, a standardised coding of HRH strengthening activities is questionable. Which code is best to capture salary support or activities related to health workforce retention? Is there a similar interpretation employed by all programme management personnel in bilateral and multilateral agencies? Our own exercise, whilst applying recognised global frameworks and based on detailed assessments of country programming, is itself an interpretation that others could question. In the absence of discrete codes for HRH or improved mechanisms to categorise this within other codes we may be resigned to accepting that this is an inherent institutional obstacle to qualifying HRH spending across programmes and development partners.

The knock-on effect of these deficiencies is considerable and has a potential impact on the efficiency of all 'aid to health'. By obscuring the volume of aid committed to HRH strengthening the global community is less informed on its share and the weight of attention that it may deserve in wider discussion on aid effectiveness, evaluation and research priorities. Discussions on governance, transparency and efficiencies of workforce investments are also stifled. In turn this could be of detriment to country plans to scale-up their health workforce and promote effective HRH management. The latter being of critical importance to the efficiency and impact of all ODA investments [36].

Lastly, we recognise that estimating ODA expenditures through imputed percentages is a model that comes with caveats and limitations. The G8's example in Table 1 to estimate expenditures on MNCH was developed with inputs from the OECD, the World Bank and the Countdown to 2015. It has a level of validation associated with these agencies. However, there remain a number of questions on their underlying assumptions. It is not for this paper to fully review these assumptions but it is suffice to recognise that the model, whilst providing a referenced framework for DFID's internal exercise on ODA for HRH, is only an exploratory first step to guide more detailed analysis. In the absence of robust data with standardised coding on HRH expenditures, the model has some utility as an initial 'yardstick'.

## Conclusions

This paper reports on an internal exercise to qualify and quantify the United Kingdom's commitments to HRH strengthening. In undertaking this process it became evident that the Agenda for Global Action could also serve a secondary function to capture bilateral and multilateral activities and investments. If applied across partners and countries it could enable a standard, comparative analysis and lead to greater synergy and alignment in future programming. Hilary Clinton's recent speech on the future of the US Global Health Initiative specifically welcomes and calls for this type of 'mapping' at country level arguing that 'there is too little innovation in capturing and understanding data' [37].

In quantifying DFID's ODA on HRH we encountered the constraints of the current CRS framework reported elsewhere in the literature. We attempted to overcome these constraints and applied a rational approach to estimating HRH investment based on new research and knowledge of the wider health portfolio. We concluded that "approximately 25%" of DFID spending in 2008/9 was for workforce strengthening. This suggests that DFID's programming on HRH is in alignment with WHO's suggested '50:50 principle'*: *where 50% of ODA should be allocated to health systems strengthening, of which at least 50% should be allocated to HRH [4]. However, the current creditor reporting system does not facilitate standardised measurement of ODA for HRH, let alone WHO's suggested advocacy to apply the '50:50 principle'.

The expression 'If you can't measure it, you can't manage it' is apt. It raises questions on the mutual accountability and managing for results elements of the Paris Declaration and how partners are responding to this [38]. The IHP+ proposed Common Framework for Monitoring Performance and Evaluating Progress in the Scale-up for Better Health states that 'the monitoring of aid effectiveness should be based upon the analysis of aid flows and information on health-system functioning' [39]. From the HRH perspective, if the independent variables on aid flows and the health system (in this case basic data on the national workforce, recurrent costs and domestic financing) provide no reliable information there is very little accountability and transparency to consider effectiveness.

This is a governance issue, above and beyond the technical interests of HRH. Further analysis within more comprehensive programmes of workforce science, surveillance and strategic intelligence will be of benefit to the aid effectiveness agenda. This will require a critical first step to address the methodological challenges in measuring donor disbursements to HRH strengthening. Without a mechanism to create and agree a baseline it will be difficult to measure progress against the calls for "more resources" and "more results" led by the United Nations Secretary General.

The Commission on Information and Accountability for Women's and Children's Health presents an opportunity to address this. It is specifically tasked to address the opportunities and challenges in using the CRS to track international development assistance to women's and children's health. By default, the Commission's remit includes ODA that is targeted to support the frontline providers of care for women and newborns - namely the health workforce. There is therefore potential for new political energy and interest from the appointed commissioners to address the limitations in reporting on ODA for HRH--or at least on ODA for the MNCH workforce, including investments in the crucial role of midwives--and to present solutions to establish a global baseline.

## Competing interests

The authors declare that they have no competing interests.

## Authors' contributions

JC conceptualised the study design and conducted the country assessments and survey. JC and IJ conducted the ODA assessment. All authors read and approved the final version.
